# Co‐coverage of reproductive, maternal, newborn and child health interventions shows wide inequalities and is associated with child nutritional outcomes in Ethiopia (2005–2019)

**DOI:** 10.1111/mcn.13452

**Published:** 2022-11-01

**Authors:** Kaleab Baye, Arnaud Laillou, Stanley Chitekwe

**Affiliations:** ^1^ Center for Food Science and Nutrition Addis Ababa University Addis Ababa Ethiopia; ^2^ Research Center for Inclusive Development in Africa (RIDA) Addis Ababa Ethiopia; ^3^ Nutrition Section UNICEF Ethiopia Addis Ababa Ethiopia

**Keywords:** child nutrition, diet, health system, maternal nutrition, nutrition‐specific interventions, stunting, wasting

## Abstract

The health system is the primary vehicle for the delivery of nutrition‐specific interventions that aim to reduce maternal and child malnutrition. The integration of nutrition interventions into existing health interventions is promising, but to ensure that no one is left behind requires that access to essential health services is equitably distributed. This study aims to assess trends and socioeconomic inequalities in coverage of reproductive, maternal, newborn and child health (RMNCH) and assess its association with child nutritional outcomes in Ethiopia. Using the Ethiopian Demographic and Health Survey (2005, 2011, 2016, and 2019), we estimated the coverage of RMNCH interventions in Ethiopia using the co‐coverage index, which is a count of the number of interventions accessed. We assessed the trend and inequalities in co‐coverage and evaluated its association with child nutritional outcomes like stunting, wasting, and minimum dietary diversity (MDD). The national co‐coverage index has shown a significant increase over the 2005–2019 period. However, all of the RMNCH interventions constituting the co‐coverage index showed a pro‐rich and pro‐urban distribution (*p* < 0.05). The highest inequality, based on the slope index of inequality (SII), was observed for skilled assistance during delivery (SII: 80.4%), followed by access to an improved source of drinking water (SII: 62.6%), and antenatal care visits (SII: 55.5%). The low coverage in RMNCH and the observed inequality were associated with stunting, wasting, and MDD. Reducing socioeconomic inequality in RMNCH is key to achieve the health, nutrition and equity‐related goals of the Sustainable Development Goals.

## Key messages

- Significant increases in the coverage of reproductive, maternal, newborn and child health (RMNCH) interventions were observed between 2005 and 2019.
- Inequalities in RMNCH interventions were stark and showed pro‐rich and pro‐urban distribution.
- Improved co‐coverage of RMNCH interventions is associated with reduced odds of stunting and wasting, and increases in dietary diversity among children 6–23 months of age.
- Analyses stratified by rural/urban residence and wealth quintile showed that the association between co‐coverage and dietary diversity was stronger for urban and the wealthiest quintile.

## Graphical abstract

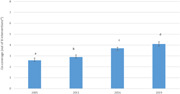

## 1 BACKGROUND

Despite substantial progress, maternal and child undernutrition remains highly prevalent in many low‐ and middle‐income countries (State of Food Insecurity, 2021). The realisation that early undernutrition can have serious short‐ and long‐term consequences have led to the integration of various maternal and child nutrition interventions into the health system (Bhutta et al., 2013). These interventions, delivered through the health system, are collectively known as nutrition‐specific interventions, and together with nutrition‐sensitive interventions that deal with the underlying factors for undernutrition, they play a critical role in preventing maternal and child undernutrition (Ruel & Alderman, 2013).

The health system has been serving as an entry point for the delivery of nutrition‐specific interventions (King et al., 2021). For example, antenatal care (ANC) and skilled‐birth attendance can provide opportunities to provide iron‐folic acid supplements, but also counsel about adequate nutrition during pregnancy and lactation, as well as advise on optimal breastfeeding and complementary feeding practices. Early childcare interventions like immunisations also pave the way for critical interventions like growth monitoring, screening for acute malnutrition, vitamin A supplementation, and so forth. These potential synergies between existing health platforms and nutrition interventions have led to the call for scaling‐up nutrition interventions delivered through the health system, but concerns over inequalities in access to health care facilities have been raised (Heidkamp et al., 2020).

In line with the Sustainable Development Goals' (SDGs) call for equitable health gains and more recent efforts to mainstream nutrition into universal health coverage (Alam et al., 2020; WHO, 2019), it is ever more critical to assess trends and socioeconomic inequalities in the coverage of reproductive, maternal, newborn and child health (RMNCH) interventions (Victora et al., 2019). Although data on the coverage of nutrition interventions is limited in multiyear nationally representative surveys like the Demographic and Health Surveys (DHS), inequalities and trends in the coverage of essential RMNCH interventions can be assessed by calculating the co‐coverage index (Heidkamp et al., 2020; Wehrmeister et al., 2016). The co‐coverage index is calculated at the mother–child pair level as the total count of essential RMNCH interventions received from a total of eight interventions representing a continuum of care. The co‐coveage index used in our study has been validated globally and was used to track progress towards universal health coverage in low‐ and middle‐income countries, including Ethiopia.

Over the last 2 decades, Ethiopia has witnessed rapid economic growth, improvements in living conditions, infrastructure and increased access to and coverage of essential health and nutrition services (Hirvonen, 2017). The introduction of the Health Extension Programme is believed to have led to significant reductions in child mortality and stunting (Lemma & Matji, 2013). However, recent studies have shown that improvements in nutritional outcomes were not equitably distributed by wealth and rural/urban residence (Baye & Kennedy, 2020; Baye et al., 2020). These inequalities could be reflections of inequitable access to health services, leading to inequitable access to nutrition services delivered through the health system. Unfortunately, whether improvements in access to basic health services are equitably distributed, and how they are associated with child stunting, wasting, and dietary diversity remains unknown due to a lack of studies.

Therefore, the present study aimed to: (i) assess the time trend in the coverage of essential RMNCH interventions in Ethiopia by socioeconomic status and rural/urban residence between 2005 and 2019 and (ii) assess the relationship between co‐coverage of RMNCH interventions and children's diet and nutritional outcomes.

## 2 METHODS

### 2.1 Overview and data source

We used the Ethiopian Demographic and Health Survey (2005, 2011, 2016, and 2019) to estimate the co‐coverage index, which served as an indicator of access to essential health services. We assessed the trend and inequalities in co‐coverage and evaluated the association between co‐coverage index and nutritional outcomes in children 6‐23 months of age including stunting, wasting and minimum dietary diversity (MDD).

### 2.2 Co‐coverage index

Co‐coverage is calculated at the mother–child pair level as the total count of interventions received from the following eight: (i) at least one ANC visit; (ii) tetanus vaccination during pregnancy; (iii) skilled birth attendant; (iv) bacillus Calmette–Guérin vaccination; (v) diphtheria, tetanus toxoid and pertussis vaccination; (vi) measles vaccination; (vii) childhood vitamin A supplementation and (viii) access to improved drinking water in the household. The co‐coverage index is a globally validated indicator used to track progress in access to health care. This index has previously been used to monitor Ethiopia's progress related to the health‐related Millennium Development Goals and is still being used to track progress towards the SDGs. For 2019, this was calculated out of seven interventions since data on tetanus vaccination during pregnancy was not available. Each mother–child pair received a score that ranged from 0 to 8/7, and all indicators were calculated for children aged 6–23 months, which is the age range for which MDD scores are validated. The 2019 DHS did not capture information on tetanus injections during the last pregnancy; hence, the co‐coverage index for 2019 was calculated out of seven possible interventions. Similarly, the pooled analyses used these seven interventions, common to all the DHS rounds (2005–2019), to calculate the co‐coverage. We used the co‐coverage cutoff points of 6 or more interventions as high, 3–5 as medium and 0–2 as low coverage, as previously described by Wehrmeister et al. (2016).

### 2.3 Inequality measures

The RMNCH interventions coverage, represented by a composite indicator (co‐coverage index) comprising essential health interventions, was disaggregated by wealth index. The wealth index in the DHS is derived from principal component analyses applied to a list of household assets/characteristics, which are country‐specific. The first quintile (Q1) represents 20% poorest families, and the last quintile (Q5) represents the 20% wealthiest families. Quintiles correspond to the relative position of households within each national sample, rather than absolute income for which data are not available for most studies.

We estimated the absolute and relative inequalities using measures recommended by Barros and Victora (2013). We calculated the percentage point differences in co‐coverage index between the wealthiest (Q5) and poorest quintiles (Q1) to estimate relative gaps between extremes in socioeconomic status. We then calculated the slope index of inequality (SII) and the concentration index (CIX), which both account for the entire socioeconomic status distribution (Q1–Q5). The SII is expressed in percentage points and CIX is a range between −1 and +1. For both SII and CIX, a value of zero represents absolute equality between the rich and the poor; whereas positive values indicate a pro‐rich distribution, and negative values indicate a pro‐poor distribution. The CIX values are then multiplied by 100 for presentation. The SII was estimated by using a regression approach, and the CIX was calculated using an analogous approach by ranking individuals according to SES position. These two measures were also used to assess whether inequalities increased or declined over time. CIX was then presented using Lorenz (concentration) curves to illustrate inequalities.

### 2.4 Statistical analyses

We estimated the prevalence (% mother–child pairs) of co‐coverage of at least one, three, and six interventions with their 95% confidence intervals for 2005, 2011, 2016, and 2019. We used the Pearson correlation coefficient (*r*) to test the crude associations between the co‐coverage index and nutritional outcomes (stunting, wasting and MDD). We then run a linear regression adjusting for residence, region and wealth as well as stratified analyses by rural/urban residence and wealth quintile. Concentration curves were used to depict inequalities by wealth and DHS rounds. Statistical analyses were conducted using Stata v14.

## 3 RESULTS

**Figure 1:**
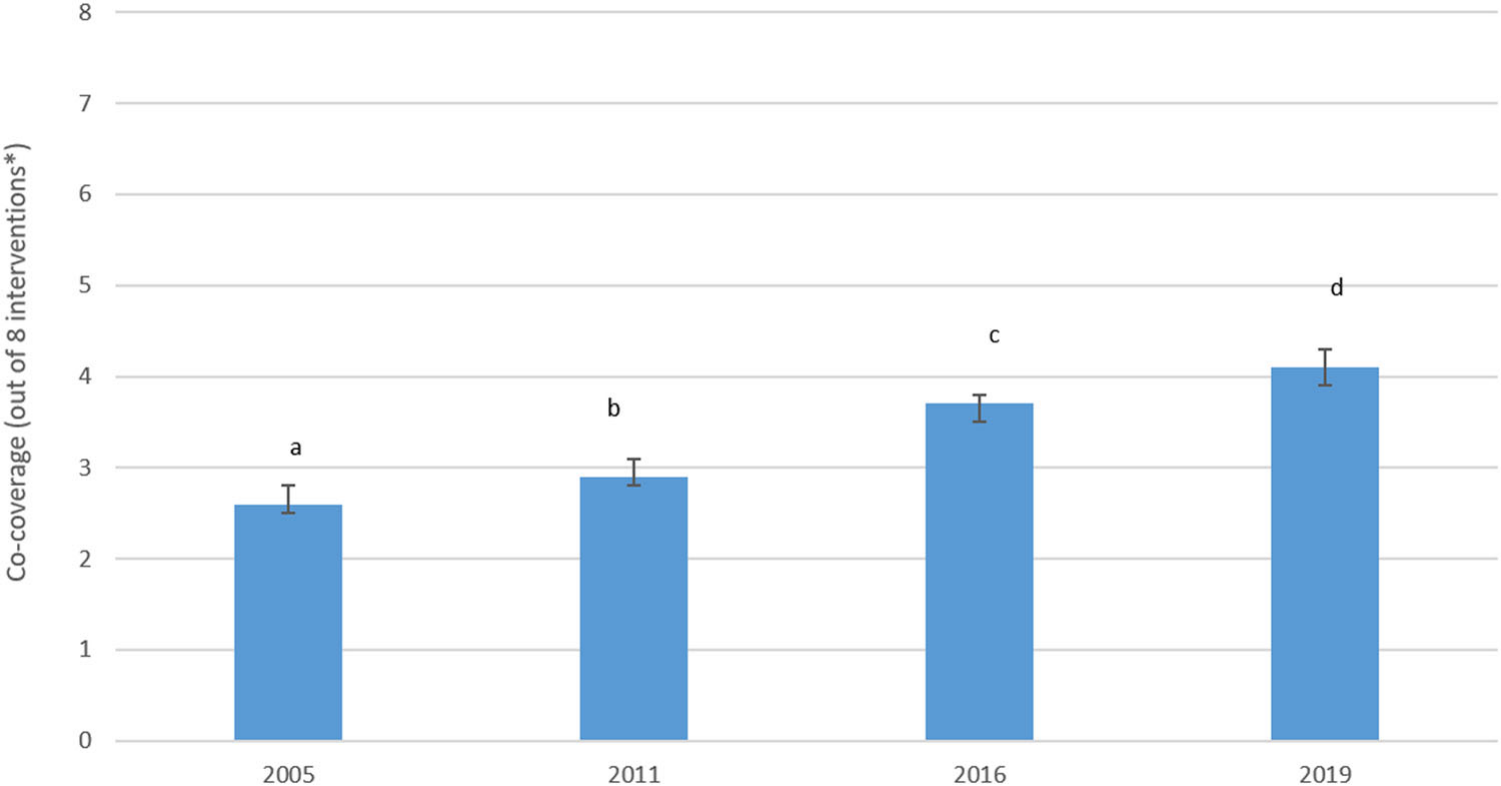
Trend in co‐coverage of reproductive, maternal, newborn and child health interventions in Ethiopia (2005–2019). *For 2019, the co‐coverage was calculated out of seven interventions.

The national co‐coverage index has shown a significant increase over the 2005–2019 period (Figure 1). The mean number of interventions accessed grew from 2.6/8 in 2005 to 4.1/7 in 2019. All eight interventions used to construct the co‐coverage index showed improvements over the years, except Vitamin A supplementation which showed some decline (Table 1). The highest change in coverage was for skilled assistance during delivery, which showed an increase of 44% points between 2005 and 2019. A 45% point increase in the coverage of women who attended at least one ANC was observed between 2005 and 2019.

**Table 1:** Trend in reproductive, maternal, newborn and child health interventions in Ethiopia (2005–2019)

	2005 (*n* = 1424)		2011 (*n* = 3022)		2016 (*n* = 3055)		2019 (*n* = 1496)	
Attended at least one antenatal care visit	31.5	(27.7, 35.6)	42.4	(39.0, 45.8)	65.4	(61.9, 68.8)	76	(71.9, 79.6)
Skilled assistance during delivery	6.8	(5.2, 8.8)	11.4	(9.6, 13.5)	35.3	(31.3, 39.5)	51	(44.8, 57.1)
BCG vaccination	58.4	(54.3, 62.4)	62.7	(58.9, 66.3)	69.8	(66.6, 72.8)	70.7	(65.5, 75.5)
DPT3 vaccination	32.6	(28.7, 36.7)	34.1	(30.9, 37.5)	52.3	(48.8, 55.7)	57.2	(51.4, 62.8)
Measles vaccination	28.4	(25.0, 32.1)	42.1	(38.9, 45.5)	44.7	(41.5, 48.0)	46.9	(41.4, 52.5)
Received vitamin A supplements in the last 6 months	49.1	(44.3, 53.9)	51.2	(48.4, 54.1)	42.7	(39.7, 45.7)	42.1	(38.0, 46.3)
Improved source of drinking water (JMP 2017)	56.4	(51.8, 60.9)	48.7	(43.9, 53.7)	58.7	(53.5, 63.8)	68	(60.8, 74.4)
Received tetanus injections during last pregnancy	40.2	(36.3, 44.1)	44.8	(41.4, 48.3)	56	(52.3, 59.5)	NA	NA

*Note*: Values are median estimates with a 95% confidence interval.

Abbreviations: BCG, bacillus Calmette–Guérin; DPT3, diphtheria–pertussis–tetanus; JMP, joint monitoring programme; NA, not applicable.

**Figure 2:**
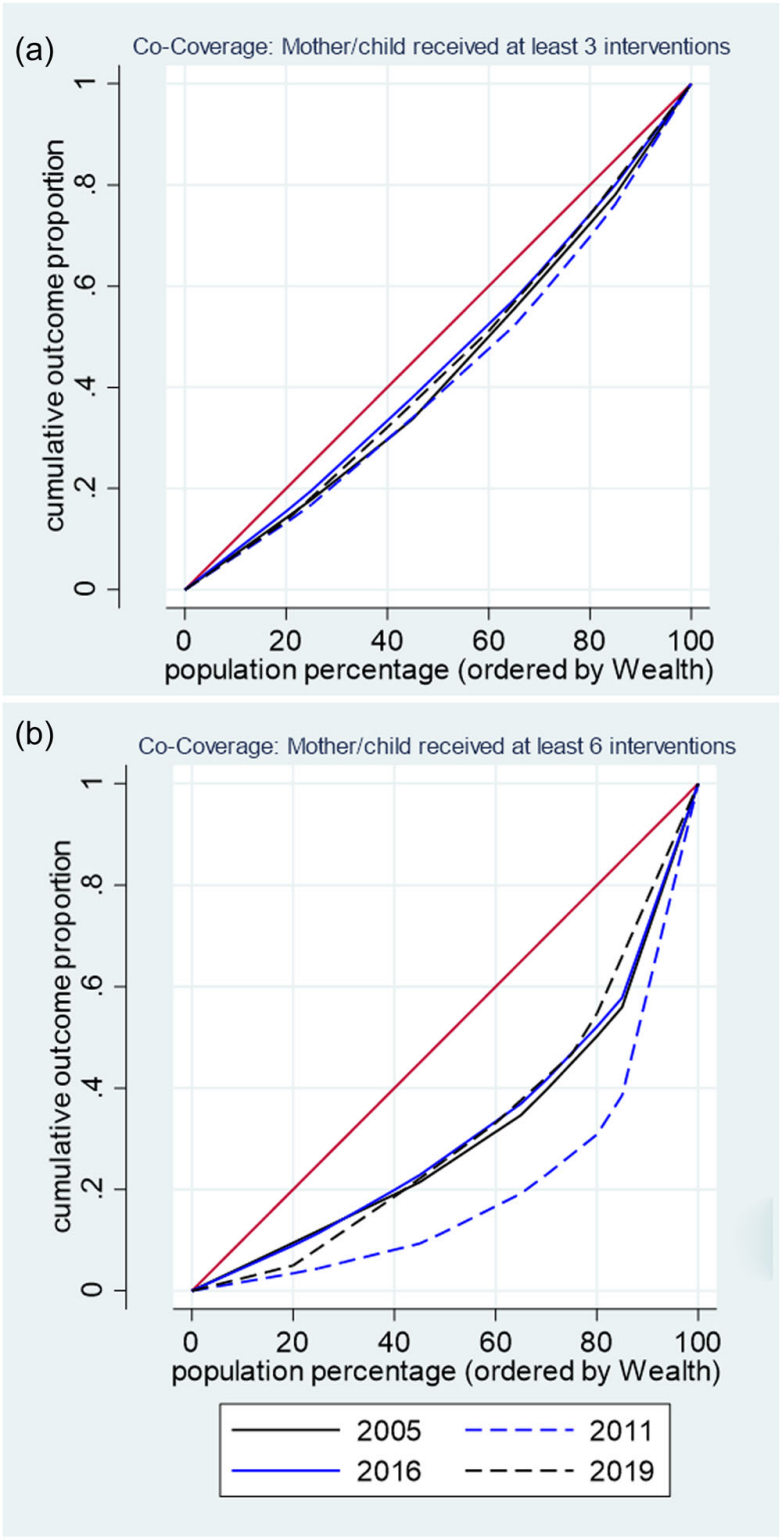
Concentration curves showing inequalities in co‐coverage index for (a) ≥3 and (b) ≥6 interventions, 2005–2019

All the RMNCH interventions constituting the co‐coverage index showed a pro‐rich distribution that was statistically significant (*p* < 0.05; Table 2). On the basis of SII, the highest inequality was for skilled assistance during delivery (SII: 80.4%), followed by access to an improved source of drinking water (SII: 62.6%), and at least one ANC visit (SII: 55.5%). This trend was also confirmed by the CIX. Although showing a pro‐rich distribution, the least inequality was observed for vitamin A supplementation. The concentration curves for at least three (≥3)—and at least six (≥6)—interventions are presented in Figure 2. While the concentration curves seemed to get closer to the equality line (diagonal line), inequalities persisted through the years as very little change was observed between the various DHS rounds. Similarly, significant differences in co‐coverage were observed between rural and urban residences (Table 3). The co‐coverage was significantly higher in urban areas (*p* < 0.001).

**Table 2:** Wealth inequality in reproductive, maternal, newborn and child health interventions in Ethiopia, 2019 (*N* = 1496)

		Q1		Q5		Slope index of inequality (SII)			Concentration index		
	National	Percent	95% CI	Percent	95% CI	SII	95% CI	*p* Value	CI	95% CI	*p* Value
Attended at least one antenatal care visit	76	47.7	[39.3, 56.1]	98	[94.4, 99.3]	55.5	[44.6, 66.4]	<0.001	11.9	[9.5, 14.3]	<0.001
Skilled assistance during delivery	51	18.7	[12.6, 26.7]	87	[73.8, 94.1]	80.4	[65.9, 94.8]	<0.001	25.7	[20.9, 30.4]	<0.001
BCG vaccination	70.7	50.2	[41.1, 59.3]	92.5	[84.0, 96.7]	48.4	[35.8, 60.9]	<0.001	11.1	[8.1, 14.1]	<0.001
DPT3 vaccination	57.2	41.1	[32.7, 50.0]	79.9	[62.4, 90.5]	43.8	[26.4, 61.2]	<0.001	12.3	[7.5, 17.2]	<0.001
Measles vaccination	46.9	35.3	[27.8, 43.6]	72.2	[59.7, 82.0]	42.7	[25.3, 60.2]	<0.001	14.6	[8.5, 20.7]	<0.001
Received vitamin A supplements in the last 6 months	42.1	35.1	[27.8, 43.3]	53.8	[47.0, 60.5]	22.5	[9.7, 35.3]	<0.001	8.6	[3.6, 13.6]	<0.001
Improved source of drinking water (JMP 2017)	68	41.2	[29.7, 53.7]	95.3	[87.9, 98.3]	62.6	[46.5, 78.8]	<0.001	15.0	[11.0, 19.0]	<0.001
Co‐coverage: Number of interventions mother/child received out of 7 (*mean*)	4.1	2.7	[2.4, 3.0]	5.7	[5.5, 6.0]	3.513	[3.021, 4.006]	<0.001	0.139	[0.119,0.159]	<0.001
Co‐coverage: Mother/child received at least three interventions	76.2	51.9	[43.2, 60.5]	98.6	[95.0, 99.6]	54.6	[44.9, 64.2]	<0.001	11.7	[9.6, 13.8]	<0.001
Co‐coverage: Mother/child received at least six interventions	29.5	7.3	[4.6, 11.6]	66.9	[58.5, 74.4]	66.6	[55.0, 78.2]	<0.001	36.1	[29.5, 42.7]	<0.001

Abbreviations: BCG, bacillus Calmette–Guérin; CI, confidence interval; DPT3, diphtheria–pertussis–tetanus; JMP, joint monitoring programme.

**Table 3:** Rural/urban inequality in reproductive, maternal, newborn and child health interventions in Ethiopia, 2019 (*N* = 1496)

	Rural		Urban		Slope index of inequality (SII)			Concentration index		
	Percent	95% CI	Percent	95% CI	SII	95% CI	*p* Value	CI	95% CI	*p* Value
Attended at least one antenatal care visit	71.1	[66.0, 75.8]	88.3	[81.8, 92.7]	39.9	[23.1, 56.7]	<0.001	4.6	[2.7, 6.5]	<0.001
Skilled assistance during delivery	42.1	[36.5, 47.9]	73.1	[54.5, 86.0]	71.7	[32.2, 111.2]	<0.001	12.4	[5.6, 19.2]	<0.001
BCG vaccination	65	[59.1, 70.4]	85.1	[71.0, 93.0]	46.7	[18.6, 74.8]	<0.001	5.8	[2.3, 9.3]	<0.001
DPT3 vaccination	52.5	[46.4, 58.5]	68.8	[54.7, 80.1]	37.6	[4.6, 70.7]	<0.001	5.8	[0.7, 10.9]	<0.001
Measles vaccination	40.0	[35.4, 44.7]	64.3	[48.4, 77.6]	56.4	[20, 92.9]	<0.001	10.6	[3.7, 17.4]	<0.001
Received vitamin A supplements in the last 6 months	38.0	[33.1, 43.2]	52.2	[45.4, 59.0]	32.9	[13.2, 52.6]	<0.001	6.9	[2.8, 11]	<0.001
Improved source of drinking water (JMP 2017)	59.9	[51.6, 67.7]	88.1	[72.6, 95.4]	65.2	[34, 96.5]	<0.001	8.5	[4.4, 12.5]	<0.001
Co‐coverage: Number of interventions mother/child received out of 7 (*mean*)	3.7	[3.4, 3.9]	5.2	[4.7, 5.6]	3.5	[2.2, 4.7]	<0.001	0.08	[0.05, 0.1]	<0.001
Co‐coverage: Mother/child received at least three interventions	70.0	[64.4, 75.0]	91.8	[85.6, 95.5]	50.6	[33.9, 67.2]	<0.001	5.8	[3.9, 7.8]	<0.001
Co‐coverage: Mother/child received at least six interventions	20.2	[16.3, 24.7]	52.8	[40.7, 64.6]	75.7	[45.7, 105.6]	<0.001	22.6	[13.7, 31.5]	<0.001

Abbreviations: BCG, bacillus Calmette–Guérin; CI, confidence interval; DPT3, diphtheria–pertussis–tetanus; JMP, joint monitoring programme.

The mean number of interventions that a child/mother was able to access was significantly associated with stunting, wasting, and MDD, even after adjusting for common covariates like wealth, rural/urban residence, and region (Table 4). The odds of stunting and wasting decreased with every 1‐unit increase in co‐coverage. On the other hand, the chance of meeting the MDD increased with increased co‐coverage. Medium (≥3; reference <3) and high (≥6 reference <6) co‐coverage were significantly associated with reduced odds of wasting, stunting and increased odds of meeting the MDD. Further stratifying the regression by rural/urban residence and wealth quintile showed that the relationship between co‐coverage and stunting was only significant for mother–child pairs from rural households (Table 5). In contrast, associations with wasting and MDD were significant for all subgroups (rural/urban and poorest/wealthiest quintile), but were stronger for the urban and the wealthiest group when higher thresholds of co‐coverage (6+ interventions) were considered. For example, having a co‐coverage of at least six interventions led to higher MDD in urban and the wealthiest quintile than in their rural and poorest counterparts (Table 5).

**Table 4:** Regression showing association between co‐coverage levels and stunting, wasting and minimum dietary diversity (MDD), 2005–2019 (*N* = 8997)

	Unadjusted			Adjusted^a^		
Stunting	*b*	95% CI	*p* Value	*b*	95% CI	*p* Value
*Co‐coverage (n out of seven)*	−0.018	−0.023, −0.014	<0.01	−0.008	−0.014, −0.002	0.008
At least one intervention	−0.044	−0.079, −0.009	0.014	−0.013	−0.049, 0.022	0.458
At least three interventions	−0.05	−0.071, −0.030	<0.01	−0.017	−0.040, 0.006	0.154
At least six interventions	−0.094	−0.118, −0.069	<0.01	−0.027	−0.056, 0.001	0.057
*Wasting*						
Co‐coverage (*n* out of seven)	−0.019	−0.023, −0.016	<0.01	−0.012	−0.017, −0.008	<0.01
At least one intervention	−0.06	−0.086, −0.033	<0.01	−0.016	−0.044, 0.012	0.258
At least three interventions	−0.069	−0.085, −0.053	<0.01	−0.037	−0.055, −0.019	<0.01
At least six interventions	−0.082	−0.101, −0.063	<0.01	−0.049	−0.071, −0.027	<0.01
*MDD*						
Co‐coverage (*n* out of seven)	0.027	0.024, 0.029	<0.01	0.017	0.013, 0.020	<0.01
At least one intervention	0.067	0.047, 0.088	<0.01	0.018	−0.002, 0.039	0.081
At least three interventions	0.08	0.068, 0.092	<0.01	0.037	0.024, 0.051	<0.01
At least six interventions	0.134	0.120, 0.148	<0.01	0.08	0.064, 0.096	<0.01

Abbreviation: CI, confidence interval.

^a^ Adjusted for residence, region, and wealth.

**Table 5:** Regression showing association between co‐coverage levels and stunting, wasting and minimum dietary diversity (MDD), stratified by residence and wealth, 2005–2019 (*N* = 8997)

	Residence^a^					Wealth quintile^b^						
	Urban (*n* = 1322)		Rural (*n* = 7675)			Poorest (*n* = 1912)			Wealthiest (*n* = 1583)			
Stunting	*b*	95% CI	*p* Value	*b*	95% CI	*p* Value	*b*	95% CI	*p* Value	*b*	95% CI	*p* Value
Co‐coverage (n out of seven)	−0.007	−0.022, 0.008	0.362	−0.008	−0.015, −0.001	0.02	0.001	−0.011, 0.013	0.87	−0.010	−0.021, 0.006	0.287
At least one intervention	−0.094	−0.358, 0.170	0.485	−0.02	−0.059, 0.020	0.332	−0.002	−0.054, 0.050	0.942	−0.030	−0.248, 0.182	0.763
At least three interventions	−0.038	−0.122, 0.047	0.382	−0.019	−0.046, 0.007	0.153	−0.003	−0.045, 0.039	0.881	−0.030	−0.107, 0.049	0.464
At least six interventions	−0.009	−0.053, 0.035	0.689	−0.029	−0.066, 0.008	0.122	0.032	−0.046, 0.111	0.417	−0.010	−0.049, 0.036	0.761
*Wasting*												
Co‐coverage (*n* out of seven)	−0.017	−0.029, −0.005	0.005	−0.011	−0.016, −0.006	<0.001	−0.02	−0.029, −0.010	<0.001	−0.020	−0.030, −0.009	<0.001
At least one intervention	−0.095	−0.388, 0.197	0.521	−0.018	−0.051, 0.015	0.289	−0.024	−0.069, 0.022	0.312	−0.050	−0.240, 0.135	0.586
At least three interventions	−0.042	−0.122, 0.037	0.299	−0.037	−0.058, −0.016	<0.001	−0.062	−0.098, −0.026	0.001	−0.080	−0.141, −0.009	0.026
At least six interventions	−0.038	−0.073, −0.004	0.028	−0.050	−0.074, −0.025	<0.001	−0.119	−0.171, −0.067	<0.001	−0.050	−0.077, −0.015	0.004
*MDD*												
Co‐coverage (*n* out of seven)	0.040	0.027, 0.054	<0.001	0.012	0.009, 0.016	<0.001	0.006	0.002, 0.010	0.002	0.040	0.029, 0.052	<0.001
At least one intervention	0.141	0.085, 0.198	<0.001	0.018	0.006, 0.030	0.004	0.016	0.006, 0.027	0.003	0.134	0.095, 0.173	<0.001
At least three interventions	0.070	0.009, 0.131	0.025	0.034	0.022, 0.047	<0.001	0.020	0.005, 0.036	0.012	0.105	0.052, 0.157	<0.001
At least six interventions	0.130	0.090, 0.170	<0.001	0.051	0.028, 0.073	<0.001	0.036	0.001, 0.072	0.045	0.122	0.083, 0.161	<0.001

Abbreviation: CI, confidence interval.

^a^ Adjusted for region and wealth; the estimate standard errors are adjusted for year and cluster.

^b^ Adjusted for region; the estimated standard errors are adjusted for year and cluster.

## 4 DISCUSSION

The present study aimed to track progress in the coverage and equity of essential RMNCH interventions over the 2005–2019 period. All the interventions assessed had a significant pro‐rich and pro‐urban distribution, suggesting that health gains were not equitably distributed, possibly leading to inequities in child nutritional outcomes.

The increase in coverage in most of the RMNCH interventions between 2005 and 2019 is promising and reflects the expanded investment in the health sector, the economic growth, and the expansion of road networks witnessed over the past decades. The national Health Sector Development Program IV (2011–2015) in Ethiopia, for example, expanded the number of primary health care facilities in rural Ethiopia (Memirie et al., 2016). Despite the bold Government's commitment to health equity, reflected in the health sector development plan I covering 2015–2020 (Federal Ministry of Health, 2015), inequalities persisted in all RMNCH interventions. The equity gap was more pronounced for maternal interventions such as ANC and skilled assistance during birth, suggesting that inequalities start before birth, and the missed opportunity to access essential interventions like iron–folic acid supplementation and nutritional counselling can contribute to adverse pregnancy outcomes, including low‐birth‐weight (Zerfu et al., 2016). Indeed, previous studies have shown that inequalities were starker for interventions that require fixed health facilities than those that can be delivered through campaigns or at the community/household level (Barros et al., 2012). Given that RMNCH interventions are provided free of charge in Governmental health facilities in Ethiopia, the inequities are likely to be primarily a reflection of inadequate access to health facilities due to remoteness, low demand for health services, and/or low‐quality health services (Baye & Hirvonen, 2020). Consequently, increasing the coverage of maternal interventions and empowering women to seek services could improve nutritional outcomes (Baye et al., 2021).

The inequalities that started with interventions during pregnancy are further exacerbated by the continued inequality in early childhood interventions (e.g. immunisations) that lead to missed opportunities for counselling on breastfeeding and complementary feeding. Combined with the unaffordability of diverse diets, this may explain the reported socioeconomic differences in the stunting and MDD prevalence in children 6–23 months of age (Baye, 2021; Baye et al., 2020). Indeed, our regression analyses adjusting for common confounders including wealth found a positive association between coverage of RMNCH interventions and MDD and an inverse relationship with stunting and wasting. This further highlights the need to increase equitable coverage of RMNCH interventions, but also the need to improve nutritional outcomes.

Beyond increasing coverage of RMNCH interventions and closing equity gaps, closing the opportunity gap by effectively integrating nutrition interventions into health services could further improve nutritional outcomes (Heidkamp et al., 2020). As RMNCH and nutrition interventions are targeting the same mothers and children, harmonising both efforts can improve maternal and child wellbeing. This implies effectively leveraging every health contact point to provide timely and quality nutrition services and counselling adapted to the local context and needs. However, this requires building the capacity of health workers, providing supportive supervision, and creating better incentives and a working environment (Baye & Hirvonen, 2020).

The present study has a number of limitations that need to be considered when interpreting our findings. Although efforts were made to analyse the temporal trend of RMNCH coverage using various rounds of DHS data, the study remains cross‐sectional; hence, findings should not imply causality. Although the co‐coverage index has the advantage of being calculated at the individual level, which allows us to explore associations with nutritional outcomes, the index is calculated only for children 6–23 months of age. Besides, the 2019 DHS did not have data on the tetanus vaccine during pregnancy and the DHS data on vitamin A supplementation is not always consistent with estimates from other sources like that of the UNICEF database.

Notwithstanding the above limitations, our study illustrates that coverage of RMNCH has been increasing, but the distribution by socioeconomic status remained pro‐rich. Inequalities were starker for maternal interventions that are provided in health facilities. The low coverage in RMNCH and the observed inequality is associated with stunting, wasting and MDD. Reducing socioeconomic inequality in RMNCH is key to achieving the health, nutrition and equity‐related goals of the SDGs.

## AUTHOR CONTRIBUTIONS

Arnaud Laillou, Kaleab Baye and Stanley Chitekwe conceived the study. Kaleab Baye analysed the data and wrote the first draft with inputs from Arnaud Laillou. All authors read and approved the final manuscript.

## Data Availability

The data that support the findings of this study are available from the Demographic and Health Survey database https://dhsprogram.com/ after registration.
